# Randomized supplementation of 4000 IU vitamin D_3_ daily vs placebo on the prevalence of anemia in advanced heart failure: the EVITA trial

**DOI:** 10.1186/s12937-017-0270-5

**Published:** 2017-08-23

**Authors:** J. B. Ernst, S. Prokop, U. Fuchs, J. Dreier, J. Kuhn, C. Knabbe, H. K. Berthold, S. Pilz, I. Gouni-Berthold, J. F. Gummert, J. Börgermann, A. Zittermann

**Affiliations:** 1 Clinic for Thoracic and Cardiovascular Surgery, Heart and Diabetes Center NRW, Ruhr University Bochum, Georgstraße 11, D-32545 Bad Oeynhausen, Germany; 2 Institute for Laboratory and Transfusion Medicine, Heart- and Diabetes Center NRW, Ruhr University Bochum, Bochum, Bad Oeynhausen Germany; 3 Department of Internal Medicine and Geriatrics, Bielefeld Evangelical Hospital (EvKB), Bielefeld, Germany; 4 Division of Endocrinology and Diabetology, Department of Internal Medicine, Medical University of Graz, Graz, Austria; 5 Polyclinic for Endocrinology, Diabetes and Preventive Medicine (PEDP), University of Cologne, Cologne, Germany

**Keywords:** Vitamin D supplementation, Hemoglobin, Anemia, 25-Hydroxyvitamin D, 1,25-Dihydroxyvitamin D, Chronic heart failure

## Abstract

**Background:**

Low 25-hydroxyvitamin D (25OHD) levels (< 75 nmol/l) are inversely associated with anemia prevalence. Since anemia and low 25OHD levels are common in patients with heart failure (HF), we aimed to investigate whether vitamin D supplementation can reduce anemia prevalence in advanced HF.

**Methods:**

EVITA (Effect of Vitamin D on Mortality in Heart Failure) is a randomized, placebo-controlled clinical trial in patients with initial 25OHD levels < 75 nmol/l. Participants received either 4000 IU vitamin D_3_ daily or a matching placebo for 36 months. A total of 172 patients (vitamin D group: *n* = 85; placebo group: *n* = 87) were investigated in this pre-specified secondary data analysis. Hemoglobin (Hb) and other hematological parameters were measured at baseline and study termination. Assessment of between-group differences in anemia prevalence and Hb concentrations was performed at study termination, while adjusting for baseline differences.

**Results:**

In the vitamin D and placebo group, baseline proportions of patients with anemia (Hb < 12.0 g/dL in females and < 13.0 g/dL in males) were 17.2% and 10.6%, respectively (*P* = 0.19). At study termination, the proportion of patients with anemia in the vitamin D and placebo groups was 32.2% and 31.8%, respectively (*P* > 0.99). There was no between-group difference in change in the Hb concentrations (− 0.04 g/dL [95%CI:-0.53 to 0.45 g/dL]; *P* = 0.87). Results regarding anemia risk and Hb concentrations were similar in the subgroup of patients with chronic kidney disease (vitamin D group: *n* = 26; placebo group: *n* = 23). Moreover, results did not differ substantially when data analysis was restricted to patients with deficient baseline 25OHD levels.

**Conclusions:**

A daily vitamin D supplement of 4000 IU did not reduce anemia prevalence in patients with advanced HF. Data challenge the clinical relevance of vitamin D supplementation to increase Hb levels.

**Trial registration:**

The study was registered at EudraCT (No. 2010–020793-42) and clinicaltrials.gov (NCT01326650).

**Electronic supplementary material:**

The online version of this article (doi:10.1186/s12937-017-0270-5) contains supplementary material, which is available to authorized users.

## Background

Both anemia and low vitamin D status (25-hydroxyvitamin D [25OHD] values <75 nmol/L) are prevalent in patients with heart failure (HF) [1–3]. The estimated prevalence of anemia in HF varies from 12% to 67% [4, 5]. This wide range is, at least in part, due to the use of inconsistent definitions of anemia and the selection of different cohorts (e.g. new-onset HF, end-stage HF) [6]. Regarding vitamin D status, the vast majority of patients have 25OHD levels < 75 nmol/L [2, 3, 7, 8] and the prevalence of deficient 25OHD levels (<30 nmol/L) varies between 28% and 66.7% [7, 8]. Compared with age-matched healthy controls, patients with HF also have lower concentrations of the active vitamin D hormone, 1,25-dihydroxyvitamin D (1,25[OH]_2_D) [9].

Epidemiological evidence suggests that both aforementioned vitamin D metabolites are inversely associated with Hb levels and anemia prevalence in patients with cardiovascular disease [10–12], including patients with HF [2]. There is also evidence from observational studies that anemia is more strongly associated with low circulating 1,25(OH)_2_D levels than with deficient circulating 25OHD levels [2, 11–13].

Results of interventional studies with vitamin D on the prevalence of anemia have been inconsistent: Early interventional studies with small numbers of patients and/or no control-groups [14–17] support the assumption of beneficial vitamin D effects on erythropoiesis and improvement of anemia. However, results of more recent randomized controlled trials (RCTs) are mixed [18–21]. While two studies showed no effect of vitamin D on Hb levels [19, 20], two other studies showed significant decreases in the required dose of erythropoietin (EPO) and erythropoiesis stimulating agents (ESA) in the vitamin D group in patients with chronic kidney disease, respectively [18–21].

In HF, anemia is an independent risk factor of morbidity [22] and mortality [23] and treatment of anemia can improve patient outcomes [24]. Aim of the present study was to investigate the effect of a daily vitamin D_3_ supplementation on the prevalence of anemia and Hb levels in patients with advanced HF and low 25OHD levels.

## Methods

### Study design and participants

**Fig. 1:**
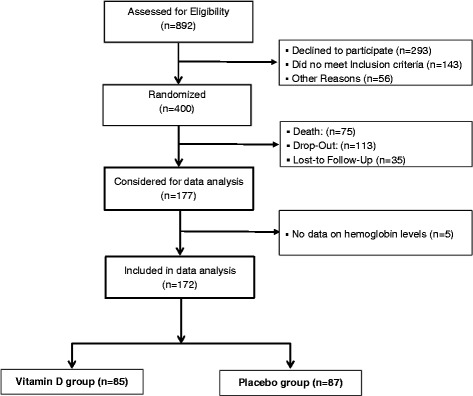
Participant flow chart of the current secondary analysis of the EVITA Trial

The present investigation is a pre-specified secondary analysis of the EVITA (Effect of Vitamin D on Mortality in Heart Failure) trial. EVITA is a single-center, randomized, placebo-controlled, clinical trial, performed at the Clinic for Thoracic and Cardiovascular Surgery of the Heart- and Diabetes Center North Rhine Westphalia, Bad Oeynhausen, Germany. Between November 2010 and July 2013, 400 patients with HF (332 men and 68 women) were recruited. All patients were ambulatory and regularly seen at our outpatient clinic. Eligible study participants were adults aged ≥ 18 to 79 years with congestive HF, New York Heart Association functional class ≥ II, and circulating 25OHD levels < 75 nmol/L. Participants were randomly allocated to receive 4000 IU (100 μg) cholecalciferol per day as oily drops (Vigantol® Oel, provider: Merck KGaA, Darmstadt, Germany) or a matching placebo (Miglyol Oel, provider: Merck KGaA, Darmstadt, Germany) for 36 months. During the study, participants remained on guideline-recommended medications. None of the study participants received erythropoietin (EPO) or iron preparations. Main study results have already been published elsewhere [25]. Of the 400 patients, 177 completed the study (Fig. 1), whereas 223 patients died, dropped out, or were lost-to follow-up. Finally, data on relevant parameters for this secondary analysis were available in 172 patients.

### Outcome measures

In the present analysis of the EVITA trial, we investigated the between-group differences in anemia prevalence at study termination. In addition, we assessed between-group differences in Hb concentrations with adjustment for baseline values. We used the World Health Organization’s gender-based definition for classifying patients as anemic (< 13 g/dL in men and < 12.0 g/dL in women).

### Biochemical measurements

Fasting venous blood samples were collected on study visits between 8 and 11 AM under standardized conditions. Blood samples were either measured directly within 4 hours of blood collection or stored at −80 °C until analysis. Circulating 25OHD (sum of 25[OH]D_2_ and 25[OH]D_3_) and 1,25(OH)_2_D (sum of 1,25[OH]_2_D_2_ and 1,25[OH]_2_D_3_) levels were measured by the autoanalyzer Liaison (DiaSorin, Stillwater, MN, USA). The measuring range for 25OHD lies between 10 and 375 nmol/l. Values < 10 nmol/l were considered 9.9 nmol/l. The limit of 1,25(OH)_2_D quantitation is 12 pmol/L and we considered values below this limit as 11 pmol/L. C-reactive protein (CRP), brain natriuretic peptide (BNP), calcium, and creatinine values were analyzed by the Architect Autoanalyzer (Abbott, Wiesbaden, Germany). Estimated glomerular filtration rate (eGFR) was calculated using the Modification of Diet in Renal Disease formula [26]. Hematological parameters such as Hb, hematocrit, erythrocytes, leukocytes, mean corpuscular volume (MCV), mean corpuscular Hb (MCH), mean corpuscular Hb concentration (MCHC), and red blood cell distribution width (RDW) were measured by automated procedures using the Abbott CellDyn 3500 hematology analyzer (Abbott, Wiesbaden, Germany).

According to published data [27], we used the following cut-off values for classifying 25OHD: < 30 nmol/l as deficient, 30–49.9 nmol/l as insufficient, and 50–74.9 as borderline. Moreover, we used earlier approaches to classify anemia hierarchically according to subtypes [10, 28]: anemia because of nutritional deficiency (iron deficiency: MCV < 80 μm^3^; folate or vitamin B12 deficiency: MCV: > 94 μm^3^), anemia of chronic kidney disease (CKD; eGFR: < 60 ml/min per 1.73 m^2^), anemia of inflammation (CRP > 10 mg/l) and unexplained anemia if none of these subtypes were present.

### Statistics

Categorical data are presented as percentages. Continuous data with a normal distribution are shown as means with standard deviation (SD) and data with a skewed distribution are shown as medians with interquartile range (IQR). Normal data distribution was checked by using the Kolmogorov-Smirnov test and was considered when probability values were > 0.05. We used the chi-squared test, unpaired *t*-test or Mann-Whitney-U-Test for baseline study group comparisons.

The McNemar test was used to assess differences in anemia status within groups. Fisher’s exact test was used to assess differences in anemia status between groups. Change from baseline data is shown as mean with 95% confidence interval [CI]. Skewed variables were log(*e*) transformed before use in parametric statistical analyses. We used ANCOVA with baseline adjustments to test for differences in the outcome variables between the two study groups at follow-up (36 months). Moreover, we used 2-factor repeated-measures ANOVA with time and study group as the between-subject variables for trend analysis in Hb concentrations. We considered *P*-values < 0.05 as statistically significant. *P*-values are two-sided. A statistical power calculation for this secondary analysis revealed a probability of 80% that the study will detect a treatment difference at a two-sided 0.05 significance level, if the true difference in Hb levels between treatments is 0.58 mg/dL. This calculation is based on 172 study participants and the assumption that the standard deviation of the Hb concentration is 1.35 mg/dL. The assumption is predicted on observational data [12] in which patients with adequate 25OHD levels had 0.6 g/dL higher Hb levels than patients with deficient 25OHD levels and their standard deviation in Hb levels was 1.4 g/dL. Statistical analyses were performed using IBM SPSS, version 21 (IBM Corp, Armonk, NY, USA).

## Results

### Baseline data

The numbers of patients who died, dropped out or were lost to follow-up in the vitamin D and placebo groups were 39 and 36, 56 and 57, and 17 and 18, respectively. Main causes of drop out were poor health condition (*n* = 30) and no pleasure to participate (*n* = 24). Baseline Hb levels were significantly higher in survivors than in nonsurvivors (14.3 ± 1.0 g/dL vs. 13.4 ± 1.2 g/dL; *P* < 0.001) but were similar in patients who dropped out and did not drop out (14.3 ± 1.1 g/dL vs. 14.2 ± 1.0 g/dL; *P* = 0.31). Baseline characteristics of the included study participants are shown in Table 1. Initial MCV was significantly lower in the vitamin D group than in the placebo group, while both were within the normal range. No significant group differences in any other clinical, biochemical, or medical treatment parameter was present (Table 1). At baseline, 17.2% and 10.6% in the vitamin D group and placebo group, respectively, had anemia (*P* = 0.19). Causes of anemia in the vitamin D and placebo group were iron deficiency in 0 and 1 patients, folate or B12 deficiency in 3 and 2 patients, CKD in 3 and 5 patients, inflammation in 0 and 0 patients, and unexplained in 3 and 7 patients, respectively. Of the 172 patients, 47.1% and 37.9% in the vitamin D group and placebo group, respectively, had vitamin D deficiency. In addition, 30.6% and 22.4% in the vitamin D group and 41.4% and 20.7% in the placebo group had insufficient and borderline 25OHD levels, respectively.

**Table 1:** Baseline characteristics of the study groups^a^

Characteristics	Vitamin D 4000 IU (*n* = 85)	Placebo (*n* = 87)	*P*-Value
Females (%)	12.9	21.8	0.12
Age (years)	56 (49–61)	54 (47–59)	0.09
Anemic subjects^b^ (%)	17.2	10.6	0.19
BMI (kg/m^2^)	28.4 (25.0–31.4)	28.1 (25.7–32.2)	0.59
NYHA (%)			
Class II	77.6	71.3	0.34
Class III	22.4	28.7	0.34
LVEF (%)	30.0 (23.5–35.0)	28.0 (24.0–35.0)	0.96
LVED (mm)	66.0 (59.0–73.0)	66.0 (60.0–75.0)	0.97
Primary Disease (%)			
Dilated cardiomyopathy	38.8	50.6	0.12
Coronary heart disease	57.6	42.5	0.05
Others	3.5	6.9	0.32
Comorbidities (%)			
Diabetes	28.2	16.1	0.06
Arterial hypertension	25.9	29.9	0.56
Renal insufficiency	10.6	8.0	0.57
Drug therapy (%)			
Loop diuretic	82.4	86.2	0.49
Thiazide diuretic	25.9	31.0	0.45
Aldosterone antagonist	81.2	86.2	0.37
ACE- inhibitor	72.9	71.3	0.81
AT II blocker	29.4	36.8	0.31
Beta-blocker	96.5	97.7	0.63
Digoxin	31.8	40.2	0.25
Antiarrhythmic drug	24.7	28.7	0.55
Lipid-lowering drug	57.6	54.0	0.63
Calcium channel blocker	3.5	3.4	0.98
Vitamin D supplement	0.0	0.0	> 0.999
Vitamin D metabolites			
25OHD (nmol/L)	30.7 (22.0–46.6)	34.4 (26.7–45.9)	0.37
1,25(OH)_2_D (pmol/L)^c^	81.6 (60.8–100.9)	87.7 (65.2–104.8)	0.18
Hematological parameters			
Hemoglobin (g/dL)	14.2 ± 1.6	14.2 ± 1.5	0.97
Hematocrit (%)	42.2 ± 4.9	42.2 ± 4.2	0.93
Leukocytes (10^9^/L)	8.1 (6.8–9.6)	7.7 (6.2–9.6)	0.37
Erythrocytes (10^12^/L)	4.7 (4.4–5.0)	4.6 (4.3–4.9)	0.42
MCV (μm^3^)	90.6 (88.1–93.4)	92.9 (90.1–95.6)	0.01
MCH (pg Hb/red blood cell)	30.7 ± 2.0	31.2 ± 2.5	0.10
MCHC (g/L)	33.6 (33.0–34.8)	33.9 (33.0–34.7)	0.87
RDW (%)	12.7 (11.9–13.5)	12.5 (11.9–13.2)	0.32
Additional parameters			
eGFR values			
≥ 60 mL/min/1.73 m^2^ (%)	69.8	73.6	0.6
< 60 mL/min/1.73 m^2^ (%)	30.2	26.4	0.62
CRP (mg/dL)^d^	0.24 (0.10–0.69)	0.21 (0.09–0.40)	0.41
BNP (pg/mL)^e^	270 (164–515)	248 (117–614)	0.96
Calcium (mmol/L)	2.39 ± 0.12	2.38 ± 0.11	0.70

*BNP* brain natriuretic peptide, *COPD* chronic obstructive pulmonary disease, *CRP* C-reactive protein, *eGFR* estimated globular filtration rate, *LVEF* left ventricular ejection fraction, *LVED* left ventricular end-diastolic diameter, *MCH* mean corpuscular hemoglobin, *MCV* mean corpuscular volume, *RDW* red blood cell distribution width

^a^median and interquartile range, mean and SD, or percentage of observations, when appropriate

^b^Hemoglobin < 12.0 g/dL in females and < 13.0 g/dL in males

^c^data are based on 161 patients

^d^data are based on 155 patients

^e^data are based on 115 patients

### Vitamin D effects

**Fig. 2:**
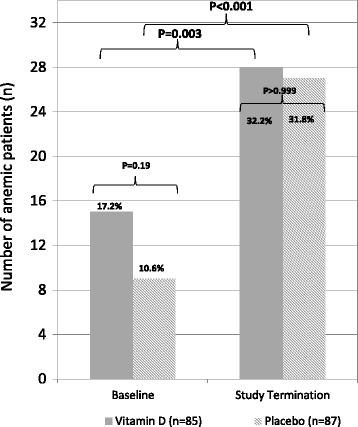
Anemia prevalence in 172 participants of the EVITA Trial at baseline and study termination; *P*-values indicate within and between study group results, when appropriate

**Fig. 3:**
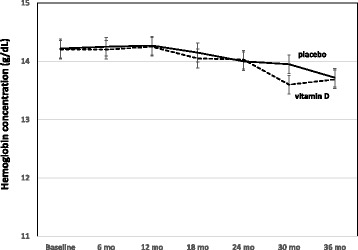
Trend analysis on hemoglobin concentrations in the vitamin D (*n* = 85) and placebo (*n* = 87) groups show significant time effects (*P* < 0.001), but no group differences (*P* = 0.91) or time x group interactions (*P* = 0.75). Data are presented as mean and 95% confidence interval

Table 2 shows the vitamin D effects on biochemical parameters. There was a significant between-group difference in circulating 25OHD levels. The increment was 55.3 nmol/L higher in the vitamin D group than in the placebo group (95% CI: 43.5 to 67.1 nmol/L; *P* < 0.001). The corresponding values of circulating 1,25(OH)_2_D were + 17.1 pmol/L (95% CI: 6.2 to 27.9 pmol/L; *P* = 0.004). Compared with placebo, vitamin D supplementation also resulted in significantly higher in-study plasma calcium levels. Prevalence of anemia increased by 15.0% in the vitamin D group (*P* = 0.003) and by 21.6% in the placebo group (*P* < 0.001; Fig. 2). No difference in anemia prevalence was determined between the two study groups at study termination (*P* > 0.99; Fig. 2). At study termination, causes of anemia in the vitamin D and placebo groups were iron deficiency in 8 and 3 patients, folate or B12 deficiency in 4 and 6 patients, CKD in 10 and 9 patients, inflammation in 2 and 2 patients, and unexplained in 4 and 7 patients, respectively. There was no significant between-group difference in change in the Hb levels (Table 2). In detail, in-study Hb levels decreased on average by −0.5 g/dL (95% CI: -0.9 to −0.2) g/dL and −0.5 g/dL (95% CI: -0.9 to −0.1) g/dL, in the vitamin D and placebo groups, respectively. The mean between-group difference in change in the Hb levels was −0.04 g/dL (95% CI: -0.53 to 0.45 g/dL; *P* = 0.87). Fig. 3 shows the trend analysis in hemoglobin concentrations by study group.

**Table 2:** Results of vitamin D treatment on biochemical parameters in patients with advanced chronic heart failure (NYHA functional class ≥ II)

Characteristics	Vitamin D group (*n* = 85)			Placebo group (*n* = 87)				
	Baseline	Follow-up (36- month)	Mean change from baseline^a^	Baseline	Follow-up (36- month)	Mean change from baseline^a^	Mean change difference between groups^a^	P-value^b^
Vitamin D metabolites								
25OHD (nmol/l)	30.7 (22.0–46.6)	92.1 (65.6–128.0)	65.6 (55.0 to 76.3)	34.4 (26.7–45.9)	40.2 (30.5–57.9)	9.9 (4.3 to 15.5)	55.3 (43.5 to 67.1)	< 0.001
1,25(OH)_2_D (pmol/l)	81.6 (60.8–100.9)	90.0 (68.3–121.8)	12.3 (3.1 to 21.4)	87.7 (65.2–104.8)	76.1 (60.5–105.1)	−7.8 (−15.5 to 0.0)	17.1 (6.2 to 27.9)	0.004
Hematological parameters								
Hemoglobin (g/dL)	14.2 ± 1.6	13.7 ± 1.8	−0.5 (−0.9 to −0.2)	14.2 ± 1.5	13.8 ± 1.9	−0.5 (−0.9 to −0.1)	−0.04 (−0.53 to 0.45)	0.87
Hematocrit (%)	42.2 ± 4.9	41.6 ± 5.7	−0.6 (−1.7 to 0.4)	42.2 ± 4.2	40.9 ± 5.6	−1.3 (−2.5 to −0.1)	0.7 (−0.8 to 2.2)	0.36
Leukocytes (10^9^/L)	8.1 (6.8–9.6)	8.2 (6.4–9.4)	−0.3 (−0.7 to 0.1)	7.7 (6.2–9.6)	7.2 (5.7–9.2)	−0.3 (−0.8 to 0.2)	0.2 (−0.4 to 0.7)	0.60
Erythrocytes (10^12^/L)	4.7 (4.4–5.0)	4.7 (4.3–5.1)	0.0 (−0.1 to 0.1)	4.6 (4.3–4.9)	4.6 (4.1–5.0)	−0.5 (−1.5 to 0.5)	0.15 (−0.04 to 0.33)	0.10
MCV (μm^3^)	90.6 (88.1–93.4)	88.7 (84.8–94.0)	−1.6 (−3.0 to −0.2)	92.9 (90.1–95.6)	90.6 (87.1–93.1)	−1.9 (−3.6 to 0.1)	0.5 (−2.4 to 1.4)	0.33
MCH (pg Hb/red blood cell)	30.7 ± 2.0	29.4 ± 2.7	−1.3 (−1.7 to 0.8)	31.2 ± 2.5	30.3 ± 2.2	−0.9 (−1.4 to 0.4)	0.6 (−1.3 to 0.0)	0.05
MCHC (g/L)	33.6 (33.0–34.8)	32.9 (31.9–34.9)	−0.8 (−1.2 to −0.4)	33.9 (33.0–34.7)	33.7 (32.8–34.5)	−0.0 (−0.4 to 0.4)	−0.7 (−1.2 to −0.3)	0.001
RDW (%)	12.7 (11.9–13.5)	13.8 (12.5–15.0)	1.0 (0.6 to 1.4)	12.5 (11.9–13.2)	13.0 (12.4–14.5)	0.8 (0.4 to 1.2)	0.2 (−0.3 to 0.8)	0.37
Additional parameters								
eGFR (mL/min/1.73 m^2^)	71.7 ± 22.5	61.9 ± 25.3	−9.7 (−13.7 to 5.8)	75.8 ± 23.4	69.9 ± 26.0	−5.9 (−10.0 to −1.8)	−4.7 (−10.2 to 0.8)	0.09
CRP (mg/dL)	0.24 (0.10–0.69)	0.27 (0.14–0.73)	0.04 (−0.12 to 0.19)	0.21 (0.09–0.40)	0.23 (0.11–0.57)	0.02 (0.0 to 0.03)	−0.08 (−0.29 to 0.13)	0.86
Calcium (mmol/L)	2.39 (2.37 to 2.39)	2.47 (2.46 to 2.48)	0.08 (0.05 to 0.11)	2.38 (2.37 to 2.39)	2.41 (2.40 to 2.42)	0.03 (0.00 to 0.06)	0.05 (0.01 to 0.10)	0.001

*1,25(OH)*_*2*_ *D* 1,25-dihydroxyvitamin D, *25OHD* 25-hydroxyvitamin D, *CRP* C-reactive protein, *eGFR* estimated globular filtration rate, *MCH* mean corpuscular hemoglobin, *MCHC* mean corpuscular hemoglobin concentration, *MCV* mean corpuscular volume, *RDW* red blood cell distribution width

^a^Results are presented as means and 95% confidence interval; ^b^probability of group differences in mean change differences between groups

### Subgroup analyses

In the subgroup of patients with chronic kidney disease (CKD) stage 3 or 4 (vitamin D group: *n* = 26; placebo group: *n* = 23) anemia prevalence at baseline was in the vitamin D and placebo groups 23.1% and 21.7%, respectively (*P* > 0.99). At study termination, anemia prevalence was 50.0% and 47.8%, respectively (*P* > 0.99). Moreover, vitamin D had no effect on mean change difference in Hb levels (0.0 g/dL [95% CI: -1.0 to 1.0 g/dL]; Additional file 1: Table S1). In addition, results did not differ substantially when data analysis was restricted to patients with baseline 25OHD levels < 30 nmol/L (data not shown).

## Discussion

The present work suggests that a daily vitamin D supplement of 4000 IU for 36 months does not reduce the prevalence of anemia in patients with advanced HF. Moreover, vitamin D had no significant effect on Hb levels. Similar results were obtained in the subgroup of patients with initial eGFR values < 60 mL/min/1.73 m^2^ and in patients with deficient baseline 25OHD levels. Obviously, hematological parameters were unaffected by the higher plasma calcium levels and the tendency for a higher risk of hypercalcemia which has been reported in those EVITA participants who were assigned to vitamin D [25].

Our data do not support results of a recent meta-analysis of observational studies [29]. This meta-analysis included seven studies with 5183 participants. Vitamin D deficiency was associated with an increased incidence of anemia (odds ratio = 2.25, 95% CI = 1.47–3.44). Our data are however in line with results of three other recent RCTs [19, 20, 30] in subjects with concurrent iron-deficiency anemia, ethnic minorities living in Norway, and hypertensive patients. In these earlier investigations, study duration ranged from 8 to 16 weeks and vitamin D doses were 400 IU and 2800 IU daily or 600,000 IU once intramuscularly. The sample sizes in these earlier studies were 30, 214, and 188 participants, respectively, and the group-differences in change in mean Hb levels were −0.8 g/dL, −0.02 g/dL and 0.04 g/dL, respectively. Although in another small, placebo-controlled trial [21] 650,000 IU vitamin D (50,000/weekly over a 4-month period) significantly decreased the required dose of EPO, no correlation between Hb levels and 25OHD concentrations could be shown. Moreover, it remains unclear why in that RCT 20 out of the 64 study subjects were excluded from data analysis. The required ESA dose was also reduced in a small RCT in children with CKD stage 5, who received vitamin D_2_ or placebo in conjunction with oral alfacalcidol (0.25 μg/capsule) for 12 weeks [18]. Unfortunately, no data on circulating 1,25(OH)_2_D levels were presented in this study. 1,25(OH)_2_D has been shown to stimulate erythroid progenitor cell proliferation via increased erythropoietin sensitivity [31]. Some earlier observational studies reported that compared with the reference category of circulating 1,25(OH)_2_D levels > 70 pmol/L, the multivariable-adjusted odds ratio for anemia was 2.35 to 4.08 in the categories of circulating 1,25(OH)_2_D levels < 40 pmol/L [2, 11, 12]. In the present RCT, the mean change difference in circulating 1,25(OH)_2_D was on average + 17.1 pmol/L. The increment is in line with a recent meta-analysis reporting that vitamin D supplements increase circulating 1,25(OH)_2_D on average by + 18.8 pmol/L (95% CI: 9.2 to 28.4 pmol/L) [32], but was probably too small to achieve significant effects on anemia prevalence. It might also be that in the present RCT the average initial 1,25(OH)_2_D level of 81.6 pmol/L was already above the threshold for stimulating erythropoiesis. It is also noteworthy that anemia risk is associated with the severity of HF [4, 23, 33, 34]. Therefore, there may simply be no causal relationship between vitamin D status and anemia in advanced HF. This assumption is further supported by the fact that even in the subgroup of patients with eGFR values of < 60 mL/min/1.73 m^2^, where EPO production and circulating 1,25(OH)_2_D levels are reduced, vitamin D treatment had no effect on anemia prevalence and Hb levels. Although vitamin D treatment decreased MCHC values, median values remained in the reference range. We therefore conclude that this vitamin D-associated between-group difference is not of clinical relevance.

Our study has several strengths, and a number of limitations. Strengths are the design of an RCT, the study duration of 3 years, and the measurement of the active form of vitamin D, 1,25(OH)_2_D, in addition to circulating 25OHD levels. One limitation is that only surrogate parameters were available on nutritional risk factors that may contribute to the development of anemia such as iron, folate or vitamin B12 deficiency, whereas data on ferritin concentrations and/or transferrin saturation for the assessment of iron deficiency were missing. Considering that iron therapy is increasingly used in HF patients [35], we have to acknowledge that previous studies suggest a suppressive effect of high-dose vitamin D on hepcidin [36], a protein with profound effects on iron metabolism including inhibition of intestinal iron absorption. However, our findings including a decrease in MCHC with vitamin D treatment strongly argue against a clinically relevant beneficial effect of a moderately high daily vitamin D dose on iron metabolism. Additional limitations are that the study population was restricted to Caucasian ethnicity and that the vast majority of patients were male. Finally, it is noteworthy that nonsurvivors had significantly lower baseline Hb levels than survivors. We therefore cannot definitively exclude the possibility that patients with lower Hb levels might have a different/more response to vitamin D supplementation compared to patients with normal or higher Hb levels.

## Conclusions

In summary, the present study found no significant effects of a daily vitamin D supplementation with 4000 IU for 36 months on the prevalence of anemia and Hb values in patients with advanced HF. Our data challenge the clinical relevance of vitamin D supplementation to increase Hb levels.

## Additional file

Additional file 1: Table S1. Results of vitamin D treatment on biochemical parameters in patients with chronic heart failure and estimated glomerular filtration rate values < 60 mL/min/1.73 m2. (DOCX 27 kb)

## Abbreviations

1,25(OH)_2_D: 1,25-Dihydroxyvitamin D
25OHD: 25-Hydroxyvitamin D
CI: Confidence Interval
CKD: Chronic Kidney Disease
EPO: Erythropoietin
ESA: Erythropoiesis Stimulating Agents
Hb: Hemoglobin
HF: Heart Failure
MCH: Mean Corpuscular Hemoglobin
MCHC: Mean Corpuscular Hemoglobin Concentration
MCV: Mean Corpuscular Volume
RCT: Randomized Controlled Trial
