# Supplementary material for: Randomized supplementation of 4000 IU vitamin D3 daily vs placebo on the prevalence of anemia in advanced heart failure: the EVITA trial
Source: Nutr J. 2017 Aug 23;16:49. doi: 10.1186/s12937-017-0270-5 (PMC5569566; doi:10.1186/s12937-017-0270-5)
Supplement: Additional file 1: Table S1. — Results of vitamin D treatment on biochemical parameters in patients with chronic heart failure and estimated glomerular filtration rate values < 60 mL/min/1.73 m2. (DOCX 27 kb) [file 12937_2017_270_MOESM1_ESM.docx]

**Table S1: Results of vitamin D treatment on biochemical parameters in patients with chronic heart failure and estimated glomerular filtration rate values <60 mL/min/1.73 m^2^**

|  | Vitamin D group (n=26) | | | Placebo group (n=23) | | |  |  |
| --- | --- | --- | --- | --- | --- | --- | --- | --- |
| Characteristics | Baseline | Follow-up (36- month) | Mean change from baseline^1^ | Baseline | Follow-up (36- month) | Mean change from baseline^1^ | Mean change difference between groups^1^ | P-value^2^ |
| **Vitamin D metabolites** |  |  |  |  |  |  |  |  |
| 25OHD (nmol/l) | 31.5 (22.9-54.2) | 79.9 (62.4-148.3) | 70.5 (45.1 to 95.9) | 34.7 (25.7-52.4) | 38.7 (30.5-63.4) | 11.8 (-1.3 to25.0) | 58.6 (29.3 to 88.0) | <0.001 |
| 1,25(OH)_2_D (pmol/l) | 74.4±41.2 | 86.5±27.9 | 12.0 (-3.7 to 27.8) | 90.3±36.6 | 68.9±38.6 | -19.2 (-36.2 to -2.1) | 21.7 (3.3 to 40.1) | 0.02 |
| **Hematological parameters** |  |  |  |  |  |  |  |  |
| Hemoglobin (g/dL) | 13.7±1.8 | 13.0±1.8 | -0.8 (-1.5 to 0.1) | 13.8±1.7 | 13.0±2.1 | -0.8 (-1.8 to 0.2) | 0.0 (-1.0 to 1.0) | 0.99 |
| Hematocrit (%) | 40.6±5.5 | 39.9±5.8 | -0.7 (-2.9 to 1.5) | 40.6±5.1 | 39.1±5.9 | -1.5 (-4.5 to 1.5) | 0.8 (-2.4 to 3.9) | 0.62 |
| Leukocytes (10^9^/L) | 8.3 (7.6-9.7) | 8.3 (6.9-9.2) | -0.5 (-1.3 to 0.3) | 8.8 (6.3-10.6) | 7.9 (5.8-9.2) | -0.8 (-1.6 to 0.1) | 0.4 (-0.5 to 1.3) | 0.40 |
| Erythrocytes (10^12^/L) | 4.5 (3.9-4.9) | 4.5 (4.1-4.9) | 0.0 (-0.2 to 0.3) | 4.4 (4.0-4.9) | 4.2 (3.8-4.6) | -2.0 (-5.8 to 1.9) | 0.3 (0.0 to 0.7) | 0.06 |
| MCV (µm^3^) | 90.6±5.2 | 88.5±8.5 | -2.1 (-5.2 to 1.0) | 93.7±6.1 | 92.3±5.0 | -1.4 (-3.8 to1.1) | -2.1 (-5.9 to 1.7) | 0.28 |
| MCH (pg Hb/red blood cell) | 31.1 (29.1-32.1) | 29.4 (26.4-31.1) | -1.9 (-2.9 to -0.9) | 32.5 (30.5-33.0) | 30.5 (28.8-31.7) | -1.2 (-2.3 to 0.1) | -1.3 (-2.7 to 0.1) | 0.06 |
| MCHC (g/L) | 33.9±1.0 | 32.5±1.4 | -1.3 (-2.1 to 0.6) | 34.0±1.3 | 33.2±1.5 | -0.8 (-1.6 to 0.0) | -0.7 (-1.5 to 0.2) | 0.13 |
| RDW (%) | 13.4±1.2 | 15.2±1.9 | 1.7 (-1.0 to 2.5) | 13.2±1.5 | 14.6±2.4 | 1.4 (-0.4 to 2.3) | 0.5 (-0.7 to 1.6) | 0.43 |
| **Additional parameters** |  |  |  |  |  |  |  |  |
| eGFR (mL/min/1.73 m^2^) | 46.0 (39.3-52.0) | 37.5 (29.8-48.0) | -5.4 (-11.3 to 0.6) | 48.0 (42.0-56.0) | 45.0 (32.0-64.0) | 0.4 (-7.2 to 7.9) | -6.3 (-15.5 to 2.9) | 0.20 |
| CRP (mg/dL)  Calcium (mmol/L) | 0.29 (0.15-0.86)  2.40 (2.39 to 2.41) | 0.33 (0.20-0.96)  2.48 (2.47 to 2.49) | -0.12 (-0.45 to 0.21)  0.06 (0.00 to 0.13) | 0.29 (0.18-0.53)  2.38 (2.37 to 2.39) | 0.41 (0.21-1.20)  2.40 (2.39 to 2.412) | 0.34 (-0.14 to 0.82)  2.41 (2.40 to 2.42) | -0.4 (-0.9 to 0.0)  0.04 (-0.06 to 0.13) | 0.23  0.12 |

^1^Results are presented as means and 95% confidence interval; ^2^probability of group differences in mean change differences between groups
1,25(OH)_2_D: 1,25-dihydroxyvitamin D; 25OHD: 25-hydroxyvitamin D; CRP: C-reactive protein; eGFR: estimated globular filtration rate; MCH: mean corpuscular hemoglobin; MCHC: mean corpuscular hemoglobin concentration; MCV: mean corpuscular volume; RDW: red blood cell distribution width
